# T Cells in Pathogenic Infections

**DOI:** 10.3390/pathogens12040578

**Published:** 2023-04-10

**Authors:** Hao-Yun Peng, Jianxun Song

**Affiliations:** 1 Department of Microbial Pathogenesis and Immunology, Texas A&M University Health Science Center, Bryan, TX 77807, USA; 2 Department of Biochemistry and Biophysics, Texas A&M University, College Station, TX 77843, USA

T cells are essential to cell-mediated immunity during bacterial, viral, and fungal infections, and immune-related diseases. Different subsets of T cells perform varying roles in safeguarding the host’s immune system. CD8 T cells use perforin and granzymes to induce apoptosis in the targeted cells. CD4 T cells orchestrate an array of immune responses, including supporting B cells in making antibodies, inducing macrophages to develop enhanced microbial activity, recruiting other immune cells to sites of infection and inflammation, and promoting CD8 T cell cytotoxicity. CD4 T cells are categorized into different subsets, including Th1, Th2, Tfh, Th17, Th22, and Treg cells. In particular, Th17 and Th22 cells exhibit dual effects on the immune system, which can either be protective or pathogenic [1,2]. The protective activity of Th17 is mainly exerted against bacterial, viral, and fungal infections through the secretion of cytokines, including IL-17, IL-21, and IL-22, at the mucosal level. On the other hand, the same pathogens can induce Th17 cells to undergo hyperactivation, which may lead to immunopathological conditions, such as rheumatoid arthritis and cancer. Th22 cells play a defensive role against pneumonia, AIDS, and influenza by (1) modulating cell fluidity and releasing cytokines and (2) protecting the epithelial barrier’s integrity and regulating cell proliferation. Conversely, in diseases such as *Helicobacter pylori* infection, hepatitis B, and SARS-CoV-2 infection, IL-22 contributes to the advancement of the disease by recruiting immune cells and inducing the release of proinflammatory cytokines in the affected area. Understanding the specific functions of different subsets of T cells and cytokines in various diseases is critical to the development of effective treatments and therapies.

Lymphocytes, including T cells and natural killer (NK) cells, play important roles in the immune response against SARS-CoV-2 infection [3]. Changes in the proportion of lymphocyte subpopulations could potentially serve as indicators of disease severity and clinical outcomes. A study by Liontos examined the correlation between lymphocyte subpopulations and clinical features in patients with severe SARS-CoV-2 infection. The researchers found that a higher proportion of NK cells was associated with an increased risk of severe lung injury, possibly indicating an overactive immune response. On the other hand, a decrease in CD4 T cells was observed to coincide with lower levels of CRP, which is a marker of inflammation, and could contribute to recovery and the maintenance of immune response balance in SARS-CoV-2 patients. CD4 T cells play a critical role in coordinating the immune response against viral infections, so a decrease in CD4 T cells could indicate an impaired immune response. However, it is important to note that the decrease in CD4 T cells observed in this study may not necessarily be the cause of lower levels of CRP or improved clinical outcomes, as there may be other factors involved. Overall, this study highlights the potential use of lymphocyte subpopulations as indicators of disease severity and clinical outcomes in SARS-CoV-2 infection. However, further research is needed to fully understand the mechanisms underlying these observations and to validate the use of lymphocyte subpopulations as diagnostic or prognostic markers.

It is common for diseases to occur concomitantly with each other. For example, major depressive disorders (MDDs) are common in individuals with long-term and systemic diseases, including tuberculosis (TB). Alvarez-Sekely and colleagues performed cytokine analysis on patients with MDD-TB, TB, and MDD, and healthy controls [4]. The frequency of IFN-γ-producing cells was higher in patients with MDD-TB compared to the other groups. Additionally, TB comorbidities with MDD were associated with reduced levels of anti-inflammatory cytokines. In addition to this insightful research article, two review articles focus on the T cell response to SARS-CoV-2 infection in individuals with comorbid conditions, such as allergies, diabetes, hypertension, and other viral infections. These articles also examine the T cell response in immunocompromised patients, including those with cancer or HIV, or who have undergone solid organ transplants [5,6]. The outcome of SARS-CoV-2 coinfections in combination with other pathogens, including viruses, bacteria, and parasites, is generally dependent on the specific pathogen involved. Wang and Peng summarized the alteration of surface markers on various T cell subsets and their associated cytokines in patients with SARS-CoV-2 comorbidities. The preliminary data suggest that immunocompromised patients exhibit considerably diminished immune responses specific to SARS-CoV-2 infection, whether they acquire it naturally or through vaccination. Therefore, Reeg emphasized that it is necessary to pay special attention to individuals with weakened immune systems in routine medical practice, as well as the development of future vaccinations. Indeed, understanding the T cell response in these high-risk populations can inform the development of targeted interventions, including personalized therapies and vaccines, to improve their outcomes. Additionally, studying the T cell response in the context of different diseases and conditions can help identify potential biomarkers for disease severity and inform clinical decision-making. Overall, ongoing research on T cells, and on their responses to diseases and comorbidities, is crucial to advancing our understanding of immune system regulation and developing effective interventions for a range of conditions.
